# Antibacterial effects of carbon dots in combination with other antimicrobial reagents

**DOI:** 10.1371/journal.pone.0185324

**Published:** 2017-09-21

**Authors:** Xiuli Dong, Mohamad Al Awak, Nicholas Tomlinson, Yongan Tang, Ya-Ping Sun, Liju Yang

**Affiliations:** 1 Biomanufacturing Research Institute and Technology Enterprise (BRITE), North Carolina Central University, Durham, North Carolina, United States of America; 2 Department of Chemistry and Laboratory for Emerging Materials and Technology, Clemson University, Clemson, South Carolina, United States of America; 3 Department of Mathematics and Physics, North Carolina Central University, Durham, North Carolina, United States of America; Massachusetts General Hospital, UNITED STATES

## Abstract

This study was designed to investigate the antimicrobial effects of CDots in combination with other antimicrobial reagents, including H_2_O_2_, Na_2_CO_3_, and AcOH (acetic acid). CDots were synthesized and passivated with 2,2’-(ethylenedioxy)bis(ethylamine) (EDA). The minimal inhibitory concentration (MIC) of CDots was 64 μg/mL on both Gram negative bacteria *E*.*coli* cells and Gram positive bacteria *Bacillus subtilis* cells. When CDots were combined with H_2_O_2_, antibacterial synergistic effects were observed based on the fractional inhibitory concentration (FIC) index, and further confirmed by an isobologram analysis and viable cell number counting methods. With the combination treatment of 10 μg/mL CDots with 8.82 mM H_2_O_2_, the viable *E*.*coli* cell numbers decreased 2.46 log, which was significant lower than the log reduction from 8.82 mM H_2_O_2_ (1.57 log) or 10 μg/mL CDots (0.14 log) treatment alone. However, the combination of CDots with Na_2_CO_3_ or AcOH did not show synergistic effects, instead, exhibiting indifference effects according to the FIC index. This study indicated that the combination of CDots with their synergistic antimicrobial reagents, such as H_2_O_2_, could reach the goal of inhibiting bacteria growth by using lower concentration of each individual chemical in the combination than using one chemical treatment alone, reduce the risks imposed on environmental health and the possibilities of the development of microbial resistances.

## Introduction

Infections with intracellular bacterial pathogens lead to the development of a number of severe diseases, presenting major challenges to our healthcare systems, from treatment needs to preventions in hospital settings and food and water supplies, and to the global public health in general [1, 2]. Especially, the rise in multidrug resistance among bacterial pathogens has threatened the effective prevention and treatment of bacterial infections, the realm of traditional antibiotics/antimicrobial agents is difficult to meet today’s society’s expectations. This has motivated a global search for alternative antimicrobial strategies, such as nanotechnology, photo-activated antimicrobial technology, and micromotor technology [3, 4]. Photo-activated antimicrobial technology is a rapidly developing field in response to the demand in development of effective control and prevention of bacterial infectious diseases. Some of the newly discovered materials and their associated technologies, particularly those may be applied in various stages prior to outbreaks of infection, have shown great potential.

Among the most recent discovered and effective photo-activated antimicrobial agents are carbon dots (CDots). CDots are small carbon nanoparticles (less than 10 nm in size) with various surface passivation schemes [5, 6], in which chemical functionalization with organic molecules has been most effective [6–8]. CDots are subject to the quantum-confinement effect [9, 10]. As a new platform of quantum dot-like fluorescent nanomaterials, the photoexcited state properties and redox processes in CDots resemble those found in conventional nanoscale semiconductors, such as the efficient photoinduced charge separation for the formation of radical anions and cations (electrons and holes) and their radiative recombinations to result in bright and colorful fluorescence emissions [6, 11, 12]. Such photo-generated electrons and holes in carbon dots can drive various catalytic processes [9, 13], and afford CDots also strong photodynamic effect [8, 14]. The same photoinduced redox processes have made CDots an excellent candidate as photo-activated antibacterial agents [15, 16] to bacterial cells, and it is particularly useful under visible/natural light illumination [15]. Other photo-activated antimicrobial agents include silver nanoparticles, titanium dioxide (TiO2), methylene blue, boluidine blue O, or combinations. For example, light-activated TiO2/Au/Mg microspheres could propel autonomously in natural water, generate highly reactive oxygen species, and efficiently destroy the cell membranes of anthrax simulant *Bacillus globigii* spores [17].

Compared to traditional antibacterial chemicals, CDots are advantageous with their known intrinsically nontoxic *in vitro* and *in vivo* [16,17,26] and environmentally benign, whereas antibacterial chemicals often pose potential toxicity to the environment, ecological systems and public health, especially in situations when high dosage is required. For example, oxidizing antimicrobial chemical agents such as hydrogen peroxide (H_2_O_2_) and sodium hypochlorite (NaOCl) are commonly used as universal disinfectants to against a wide range of microbes for more than 100 years *(12)*. However, some species of bacteria are resistant to the antiseptic action of such oxidizing chemicals and require high concentrations at which extensive damage to biological systems (human tissues) may be caused *(13)*. As a promising strategy in the antimicrobial field, combination of different antimicrobial agents is a feasible practice to achieve the maximal antibacterial activity at the minimal dose of individual agents, which effectively reduces the potential toxicity posed by antimicrobial chemicals. The combination treatments often generate synergistic or enhanced effect from the combination of different action mechanisms, or from the new effective antimicrobial molecules generated on-site. Such combination strategies have been in practice with positive outcomes by combining different antimicrobial chemicals or chemicals or nanomaterials. For example, Fenton reagent, which combined hydrogen peroxide or ascorbic acid with various metal ions together in order to generate highly reactive hydroxyl radicals in the immediate vicinity of their targets *(15–17)*, has been reported as an effective sporicidal reagent to *Bacillus* spores *(14)*. Our group has reported that carbon nanotubes (CNTs) in combination with H_2_O_2_ and NaOCl exhibited enhanced antimicrobial to bacterial cells and spores. The synergistic antimicrobial effect was observed on the combination Ag-CNTs and an antimicrobial peptide-nisin. With this regard, this study aimed to investigate whether the combination of the newly discovered photo-activated CDots with other traditional antimicrobial chemicals, including H_2_O_2_, CH_3_CH_2_COOH (AcOH), and Na_2_CO_3_, would generate synergistic or enhanced antimicrobial effect against bacterial cells.

## Materials and methods

### Preparation of CDots

The synthesis procedure of the CDots was the same as the one published previously [18]. Briefly, the commercially acquired carbon nano-powders (US Research Nanomaterials, Inc.) at the weight of 2 g was refluxed in aqueous nitric acid (8 M, 200 mL) for 48 h, then centrifuged at 1,000g to remove the supernatant. The residue was suspended in DI-H_2_O, dialyzed in a membrane tubing (molecular weight cut-off ~500) against fresh water for 48 h, and then centrifuged at 1,000 g. Upon the removal of water, small carbon nanoparticles were collected and then refluxed in neat thionyl chloride for 12h. After the removal of thionyl chloride, the sample was mixed with dried 2, 2’-(ethylenedioxy)bis(ethylamine) (EDA, Sigma-Aldrich) liquid, heated to 120°C, and vigorously stirred under nitrogen protection for 3 days. The reaction mixture was cooled to room temperature, dispersed in DI-H_2_O, and then centrifuged at 20,000 g to retain the supernatant. It was dialyzed in a membrane tubing (cutoff molecular weight ~500) against fresh water to remove unreacted EDA and other small molecular species to obtain EDA-CDots as an aqueous solution. The EDA-CDots were characterized by using NMR, microscopy, and optical spectroscopy techniques in a previously published paper [19]. The size of EDA-CDots was 4–5 nm in average diameter.

### Evaluation of inhibitory effects of CDots, H_2_O_2_, Na_2_CO_3_, and AcOH on bacterial growth by optical density (OD) measurement

Overnight grown *E*. *coli* or *B*. *subtilis* cells, which were kindly provided by Dr. Jiahua Xie, were diluted with LB broth to the concentration of 1×10^6^ cells/mL. The bacterial growth measurement was performed in 96-well plates. Each well contained 100 μL bacteria cells with various concentrations of CDots, H_2_O_2_, Na_2_CO_3_, or AcOH in the final volume of 150μL. The samples were then exposed to white light for 1 h by the use of light box (Arbor Scientific, MI) on a shaker (Lab-Line Instruments, Inc, IL) at the setting of 2. The treated bacterial samples were then incubated at 37°C for 24 h. The optical densities (OD) of the samples were measured at wavelength 595 nm before and after incubation using SpectraMax M5 multi-detection reader with the software SoftMax Pro5.4.5 (Molecular Devices Corp., CA). The magnitudes of increase in OD595 values indicated a measure of bacterial growth. Inhibitory effect of treatment on bacterial growth can be evaluated by the less percentage of OD595 value compared to the untreated control samples.

### Microdilution checkerboard method to determine FIC index of the combination treatment with CDots and H_2_O_2_, Na_2_CO_3_, or AcOH

To investigate whether there were synergistic antimicrobial effects in the combination treatment with CDots and H_2_O_2_, Na_2_CO_3_, or AcOH, FIC indexes were determined by the use of broth microdilution checkerboard method according to previous publications [20–23]. Using the combination treatment with CDots and H_2_O_2_ as an example, the experiments were designed as following. In a 96-well plate, aliquots of 100μL *of E*. *coli* or *B*. *subtilis* cells (1.0×10^6^/mL) were distributed into each well, CDots and H_2_O_2_ solutions at various concentrations were added to the wells to achieve 2-fold serial dilutions along the ordinate and the abscissa of the plates, respectively. The final volume in each well was adjusted to 150 μL with DI-H_2_O. The resulting checkerboard contained each combination of CDots and H_2_O_2_. The samples were exposed to visible light from a 12V 36W light bulb (at a distance of 10 cm) for 1 h, and then incubated at 37°C for 24 h. OD595 values were measured before and after incubation. The increase of OD595 value after incubation indicated bacteria growth. The minimal inhibitory concentration (MICs) of each agent was defined as the lowest concentration that completely inhibited the bacterial growth. In the combination treatments, the fractional inhibitory concentration (FIC) index (ΣFIC) was calculated according to the equation: ΣFIC = FIC of agent A + FIC of agent B, where FIC of agent A = MIC of agent A in combination/MIC of agent A alone, and FIC of agent B = MIC of agent B in combination/MIC of agent B alone. The antimicrobial effects of the combination treatment was determined by the resulting ΣFIC as follows: ΣFIC ≤ 0.5 indicates synergy; 0.5 < ΣFIC < 1.0 indicates partial synergy; ΣFIC = 1.0 indicates additive; 1.0 < ΣFIC < 4.0 indicates indifference; and ΣFIC ≥ 4.0 indicates antagonism [24, 25].

### Isobologram analysis for synergistic effect

To graphically visualize the interactions between CDots and H_2_O_2_ within the combination treatment, an isobologram was generated by plotting the FIC values on the X-Y coordinate, using the concentrations of CDots and H_2_O_2_ as the x- and y-axis, respectively. The MIC of each reagent was then plotted on the graph and the two data points were joined by a line. The MICs of the combined reagents were plotted and joined by another line and compared with the previous line. The interaction between the two reagents is considered synergistic if the line of the combined reagents’ MICs lies below the line of individual MICs, vice versa, the interaction is antagonistic. If the line is at the same position as the previous line, then there is no interaction between the two reagents [21, 26, 27].

### Determination of viable cell numbers after CDots treatment alone or in combination with H_2_O_2_, Na_2_CO_3_, or AcOH

A freshly grown overnight *E*. *coli* or *B*. *subtilis* culture was diluted to the concentration of ~ 1×10^6^/mL in PBS. Cells were then treated with CDots, H_2_O_2_, Na_2_CO_3_, or AcOH alone at various concentrations, or with the combination of 10 μg/mL CDs with H_2_O_2_, Na_2_CO_3_, or AcOH at various concentrations. The treatments were performed in 96-well plates. Each well included 150 μL cells, antimicrobial reagents, and DI-H_2_O to reach the final volume of 200 μL. Similar as the FIC test, the samples were exposed to visible light with constantly shaking at room temperature for 1 h. Each sample was 10-fold serial diluted with PBS, and then the appropriate dilutions were plated on LB agar plates. Colony numbers were counted after 18 hours growth at 37°C, then the viable cell number of each sample was calculated in colony forming unit per mL (CFU/mL). The reduction of viable cell number after the treatment was used to evaluate and compare the antimicrobial effect of each treatment.

### Scanning electron microscopy (SEM) imaging

Fresh grown *E*.*coli* cells were washed three times and resuspended in PBS solution. The cells were then treated with 8.82 mM H_2_O_2_ and 10 μg/mL CDots alone or in combination under light for 1 h, followed by overnight fixation with 4% formaldedyde and 2% glutaraldehyde solution at 4°C. After removing fixative and washing with DI-H_2_O, the samples at the volume of 10 μL for each were loaded on silicon slide covers and air-dried. All the samples were then coated with gold using Denton Vacuum Desk IV (Czech Republic). SEM images were taken using the FEI XL30 microscope (Netherlands) at the Shared Materials and Instrumentation Facility (SMIF) at Duke University.

### Statistical analyses

Statistical analyses were performed by using the general linear model (GLM) procedure of the SAS System 9.2 (SAS Institute Inc., Cary, NC, USA), with P<0.05 being considered as significant different.

## Results and discussion

### Minimal inhibition concentration of CDots to inhibit *E*. *coli* and *B*. *subtilis* growth

**Fig 1:**
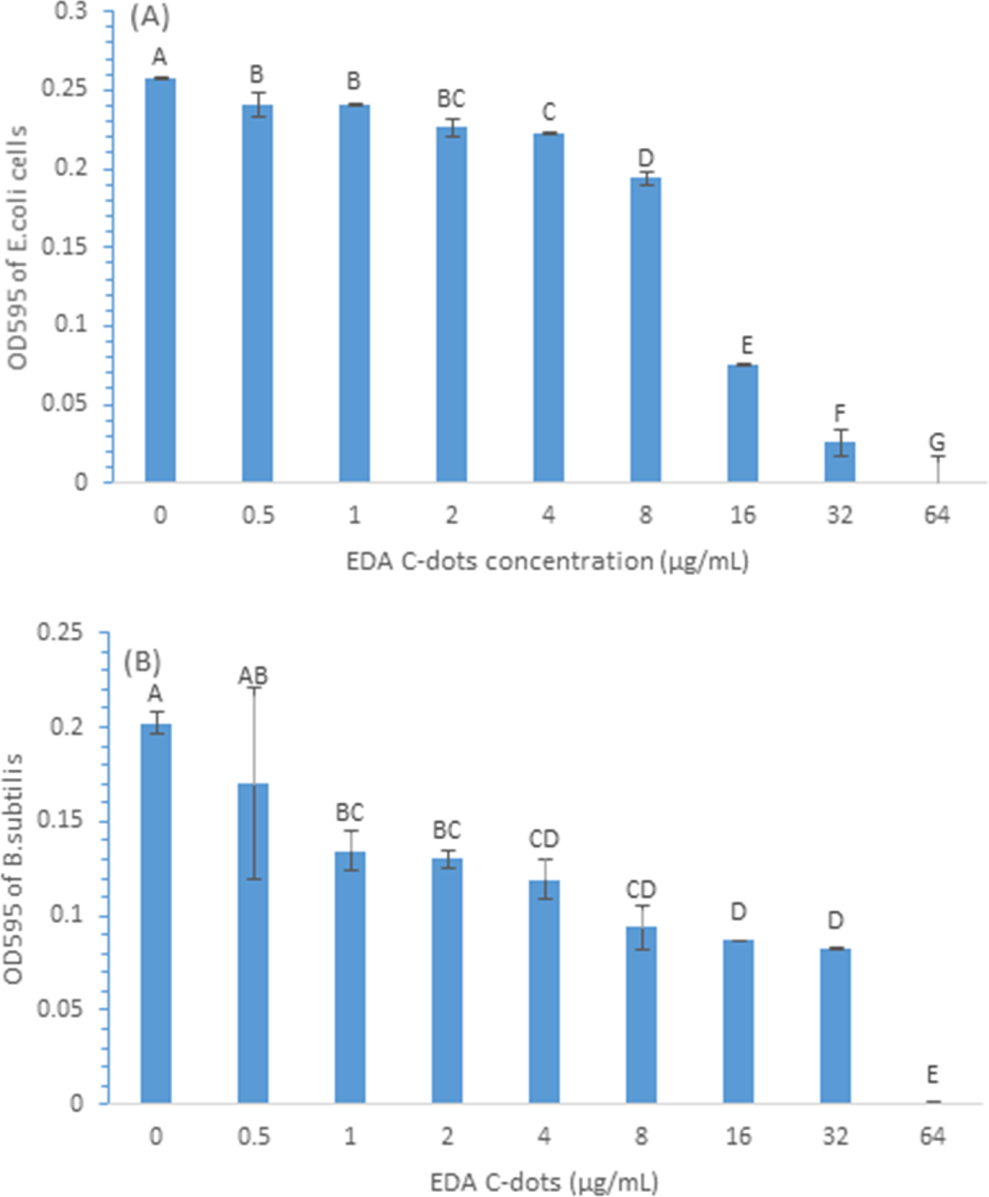
Minimum inhibitory concentration (MIC) of EDA-CDots on *E*. *coli* (A) and *B*. *subtilis* cells (B) after 24 h at 37°C. Data is presented as the mean values with ±SD as Error bars. Different letters above the columns indicate statistically significant differences (p<0.05).

Fig 1 shows the growth *E*. *coli* (A) and *B*. *subtilis* (B) measured by OD after the cells were treated with a range of CDots for 1 h under visible light illumination and followed by incubation in the presence of the CDots for 24 h. As shown in the Fig 1A and 1B, CDots inhibited the growth of both Gram negative bacteria *E*. *coli* cells and Gram positive bacteria *B*. *subtilis* cells, and the inhibitory effect was CDots concentration dependent. With the concentration of CDots increased from 0.5 to 64 μg/mL, CDots exhibited increasing inhibitory effect on both Gram negative bacteria *E*. *coli* cells and on Gram positive bacteria *B*. *subtilis* cells in similar trends, with slight variations to the two types of cells at each given concentration, and the MIC of CDots to completely inhibit the growth of both types of cells was 64 μg/mL.

The current mechanistic framework for the known optical properties of CDs is such that upon photoexcitation there are efficient charge separations for the formation of radical anions and cations (electrons and holes in a somewhat different description), which are “trapped” at various passivated surface sites. The redox species and emissive excited states could lead to the bactericidal functions [28], which could be similar to traditional quantum dots when on irradiation, the dots transfer energy directly to molecular oxygen to generate singlet oxygen [29].

### Synergistic effect of the combination treatment with CDots and H_2_O_2_

**Fig 2:**
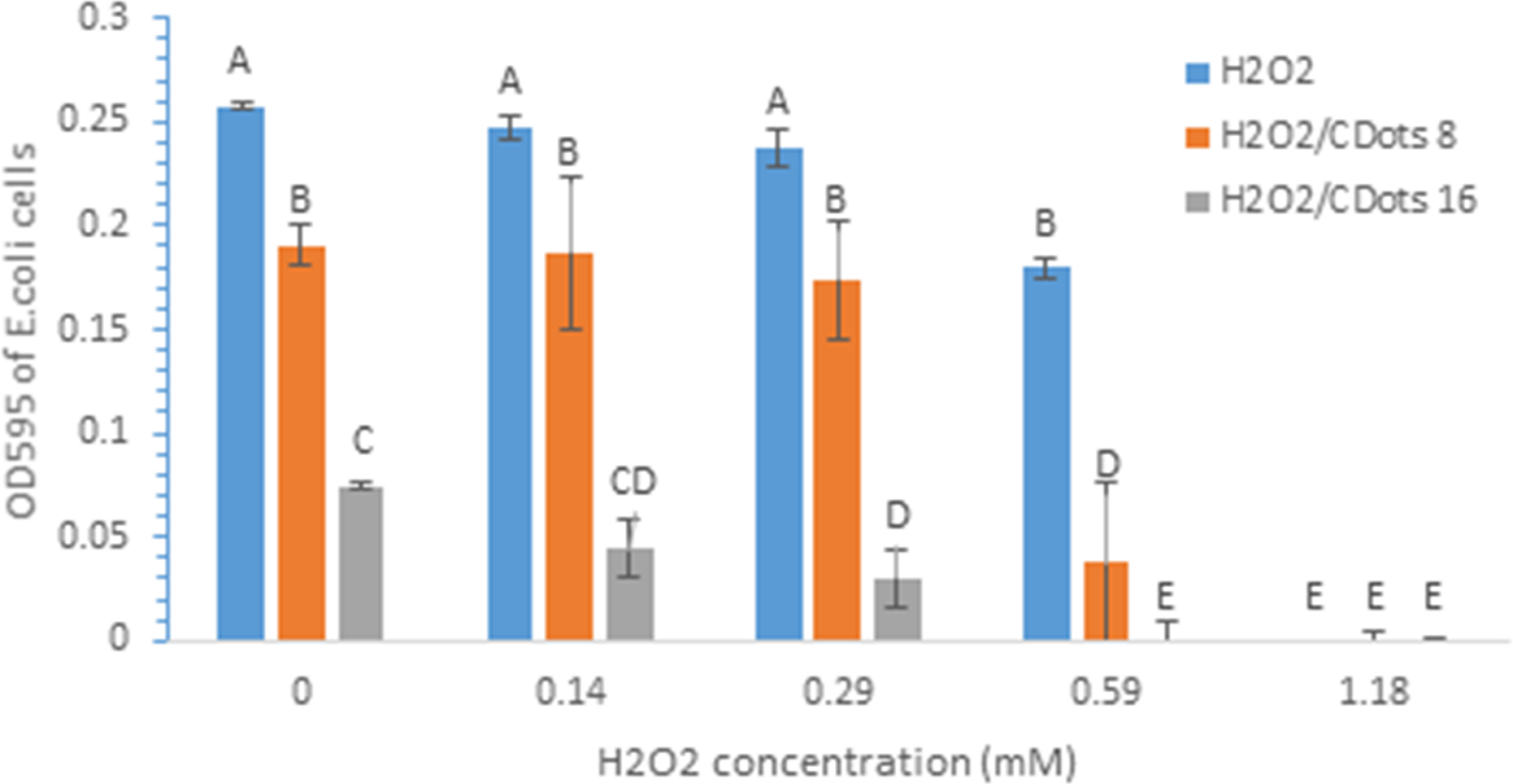
Inhibitory effects of H_2_O_2_ alone and H_2_O_2_/CDots combination on *E*. *coli* cells. CDots concentration was 8 μg/mL or 16 μg/mL. Bacterial cell growth was measured by OD value at wavelength 595 nm. Data is presented by the mean of 3–5 replicated samples and Error bars are ±SD of the replicated measurements. Different letters above the columns indicate statistically significant differences (p<0.05).

We first examined the minimal inhibitory concentration of H_2_O_2_ alone and the combination of H_2_O_2_ and two different concentrations of CDots to the growth of *E*.*coli* cells. Fig 2 shows the growth of *E*. *coli* cells measured by OD at 24 h of incubation after the cells were treated by H_2_O_2_ alone and the treatments combining H_2_O_2_ with 8 or16 μg/mL CDots. Obviously, H_2_O_2_ alone exhibited strong inhibitory effect on the growth of *E*. *coli* cells. The MIC of H_2_O_2_ alone to *E*. *coli* cells was 1.18 mM. However, when H_2_O_2_ was combined with 8μg/mL or 16 μg/mL CDots, the inhibitory effects were significantly enhanced (p<0.05). For instance, when *E*. *coli* cells were treated with 0.59 mM H_2_O_2_ alone or 8 μg/mL CDots alone, the growth of cells only decreased by ~30% and ~26%, respectively; when the cells were treated by 0.59 mM H_2_O_2_ combined with 8 μg/mL CDots, the cell growth decreased by ~88% decrease (OD value decreased from 0.26 to 0.03). The combination of 0.59 mM H_2_O_2_ with 16μg/mL CDots completely inhibited *E*. *coli* cell growth. Similar trends were also observed on *B*. *subtilis* cells.

To further investigate whether there was synergistic inhibitory effect of the combination treatment of H_2_O_2_ with CDots to *E*. *coli* cells, the microdilution checkerboard method was used, the minimal inhibitory concentrations (MICs) of individual agents and combined treatments were determined, then the ΣFIC for each combination treatment was obtained (Table 1). As shown in Table 1, MICs of CDots and H_2_O_2_ were 64 ug/mL and 1.18 mM on *E*. *coli* cells, respectively. When CDots at the concentration of 32, 16 or 8 μg/mL were combined with H_2_O_2_, the FIC index were between 0.5 or 0.625. These ΣFIC values indicated that the combination of CDots with H_2_O_2_ had partial synergistic inhibition effects on *E*. *coli* cells.

**Table 1:** The MICs and FICs of individual agents in the combination treatments of CDots with H_2_O_2_, and the total FICs of combination treatments, obtained using the microdilution checkerboard method.

Combination Treatments		FIC of CDots	FIC of H_2_O_2_	ΣFIC
CDots (μg/mL)	H_2_O_2_ (mM)			
64 (MIC)	0	-	-	-
32	0.037	0.5	0.03125	0.53125
32	0.073	0.5	0.0625	0.5625
32	0.147	0.5	0.125	0.625
16	0.294	0.25	0.25	0.5
8	0.588	0.125	0.5	0.625
0	1.176 (MIC)	-	-	-

**Fig 3:**
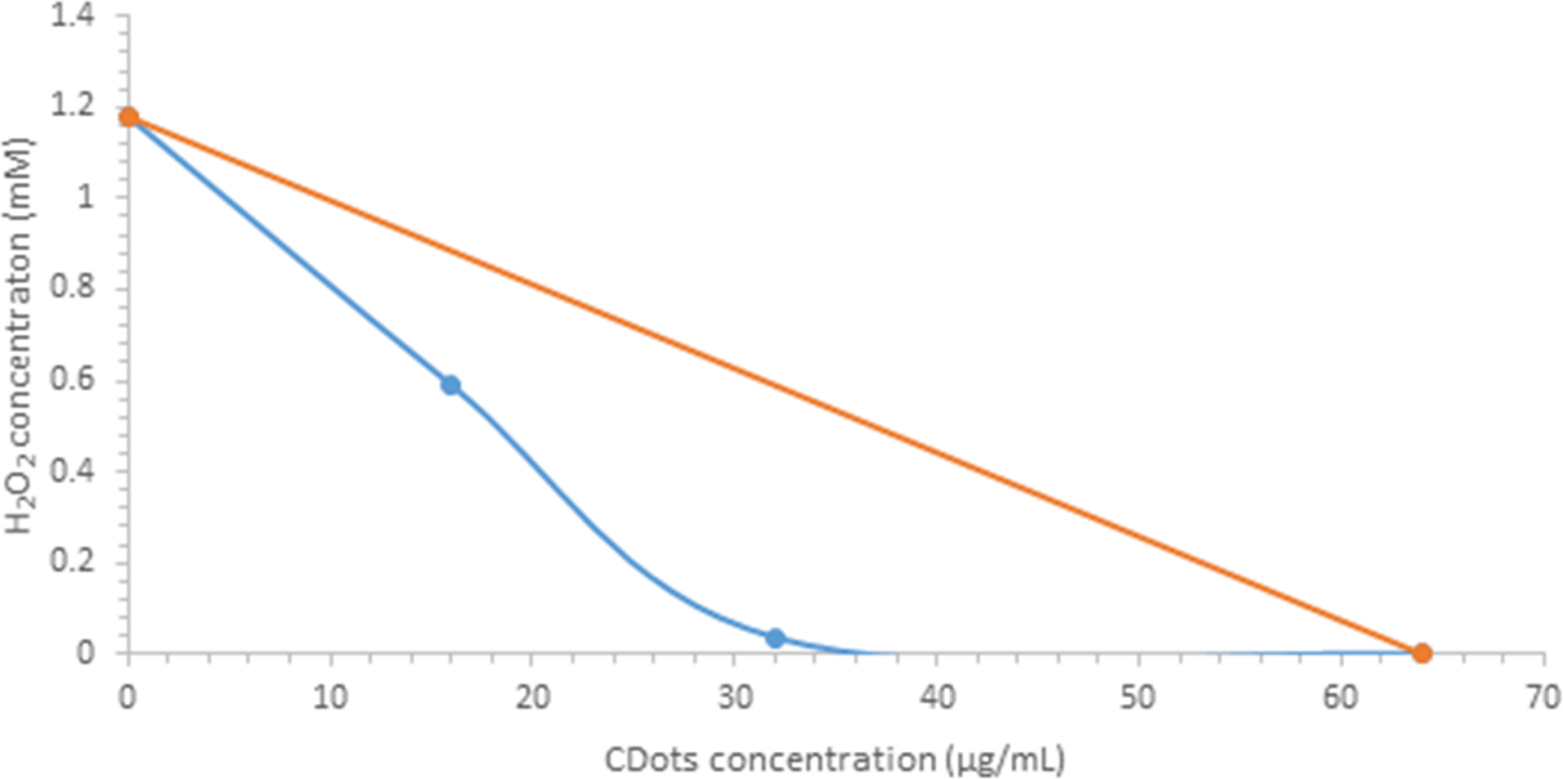
Isobologram of the interaction between CDs and H_2_O_2_ against *E*.*coli* cells.

The synergistic effect of the combination treatment with CDots and H_2_O_2_ to *E*. *coli* cells was further proved by isobologram analysis. Fig 3 shows the isobologram of the combination treatment with H_2_O_2_ and CDots, where the line of MICs in the combination treatment lies below the line linking the MICs of individual H_2_O_2_ and CDots, indicating a synergistic effect between H_2_O_2_ and CDots.

**Fig 4:**
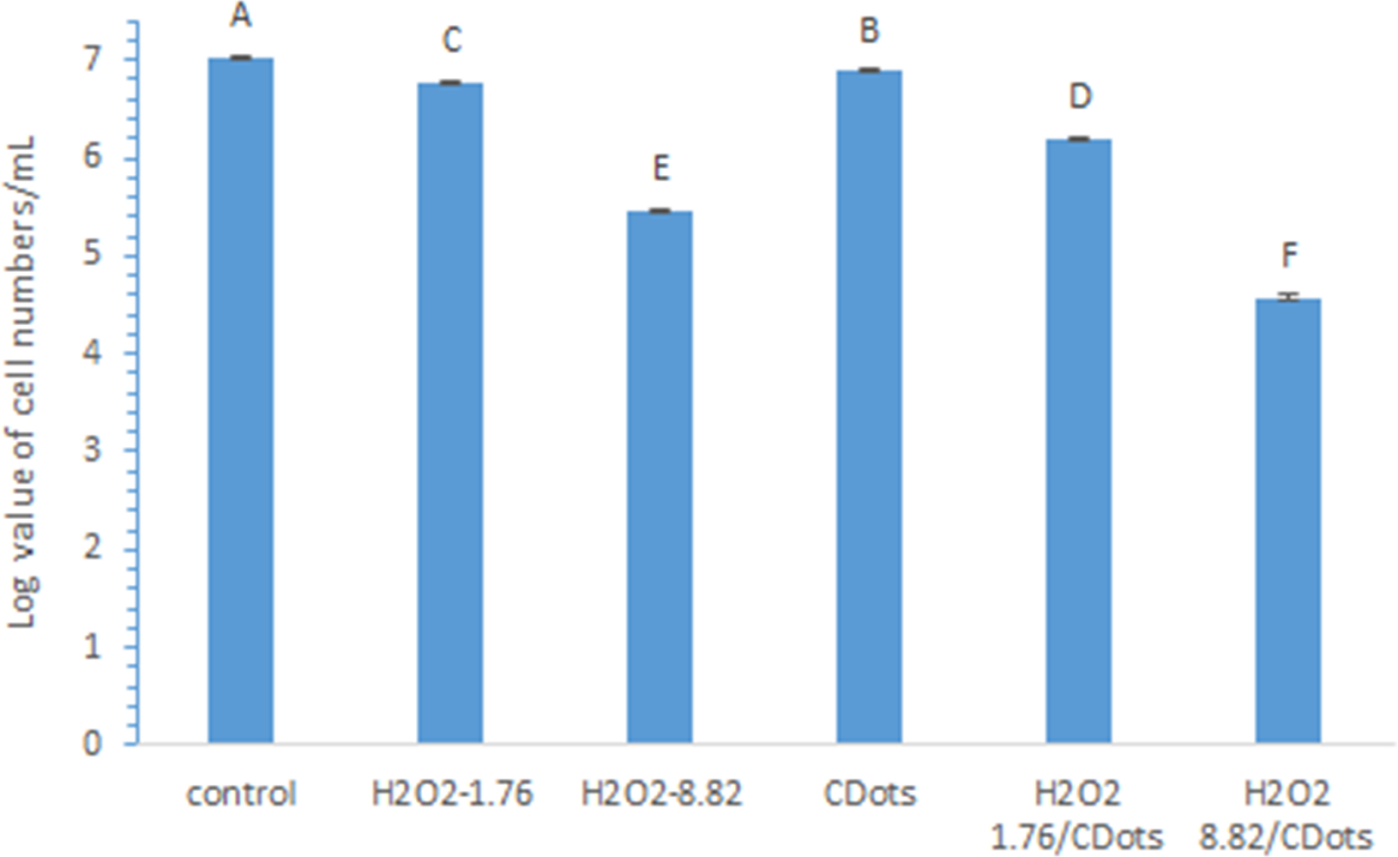
Viable *E*.*coli* cell numbers after treated with 1.76 mM H_2_O_2_, 8.82 mM H_2_O_2_, or 10μg/mL CDots alone or H_2_O_2_/CDots combination. Different letters above the columns indicate statistically significant differences (p<0.05).

In a separate experiment, the treatments with individual H_2_O_2_ or CDots, and the combination treatments of H_2_O_2_ with CDots were applied to *E*.*coli* samples containing as high as ~10^7^ CFU/ml cells, then the viable cell reduction in the samples were determined after the treatments. The comparison between the viable bacterial cell numbers after the cells treated with 10 μg/mL CDots alone or 1.76 mM or 8.82 mM H_2_O_2_ alone for 1 h, and cells treated with the combination treatment of 1.76 mM H_2_O_2_, or 8.82 mM H_2_O_2_ with 10 μg/mL CDots for 1 h, also provided quantitative evidence for the synergistic effect. As shown in Fig 4, the treatment by 1.76 mM H_2_O_2_, 8.82 mM H_2_O_2_, or 10 μg/mL CDots alone resulted in viable cell reduction of 0.26log, 1.57log, and 0.14log, respectively (Fig 4). The viable cell reduction resulted from the combination of 1.76 mM H_2_O_2_/10 μg/mL CDots and the combination 8.82 mM H_2_O_2_/10 μg/mL CDots, was 0.84 and 2.46, respectively. The results indicated that the viable cell reductions resulted from the combination treatments were greater than the sum of the treatments with individual component, which again confirmed the synergistic effect of H_2_O_2_/CDots combination treatment. This synergistic effect was also confirmed by *E*.*coli* cell growth curves (S1 Fig). The untreated control sample, CDots alone treated sample, and H_2_O_2_ alone treated sample reached OD595 value of 0.15 at the growth time 3.3, 3.5, and 4 h, respectively, whereas the H_2_O_2_/CDots treated sample did not grow even after 8 h incubation.

The synergistic effect of the combination of H_2_O_2_ and CDots is apparently from the combination of multiple action mechanisms from the individual components in the way one mechanism is enhanced by others, which provide the antibacterial activity by damaging the microorganisms in different ways [30]. Such combination treatments were able to achieve the bacteria inactivation at lower concentration of each component than the individual treatments by each component, thus reduced potential toxicity posed to environment or health. Due to the more complicacy of antimicrobial mechanisms, the combination treatments also reduced microbial resistance development. In the combination of H_2_O_2_ and CDots, H_2_O_2_ by mechanism is known to produce hydroxyl free radicals, which are highly reactive and attack DNA, RNA, proteins, and lipids in microbes [31]. H_2_O_2_ also acts as one of the strongest oxidizers and effective against all forms of microorganisms. CDots in current mechanistic framework for the known optical properties is such that upon photoexcitation there are efficient charge separations for the formation of radical anions and cations (electrons and holes), which are “trapped” at various passivated surface sites. The radiative recombination of redox pairs is responsible for the observed fluorescence emissions, and in principle, might be responsible for the observed bactericidal functions. The synergistic antimicrobial effect of the combination of CDs and H_2_O_2_ was mostly likely acting in the way that the mechanism of H_2_O_2_ and CDots enhanced each other.

The synergistic effect of H_2_O_2_ with other nanomaterials, or nanomaterials with organic dyes, was previously reported. In our previous study, the combination treatment of Ag-CNT and H_2_O_2_ showed a synergistic effect on the inactivation of *E*. *coli* cells [21]. In a study reported by Narband et al. [32], the combination of CdSe/ZnS quantum dots with toluidine blue O (TBO) at the ratio of 1: 2667 exhibited synergistic effect against bacterial cells, as the quantum dots acted as an enhancer to the light-activated TBO. Sun et al. [4] combined graphene quantum dots (GQDs) with a low level of H_2_O_2_ and found that GQDs significantly enhanced antibacterial activity of H_2_O_2_. It was known that GQDs show the peroxidase-like activity and can catalyze the decomposition of H_2_O_2_, generating ∙OH. The creation of ∙OH from H_2_O_2_ improves the antibacterial performance of H_2_O_2_ since ∙OH has a higher antibacterial activity [4]. Unlike GQDs, although CDots and H_2_O_2_ showed synergistic antimicrobial effects, there was no available literature reported the peroxidase-like activity of CDots.

**Fig 5:**
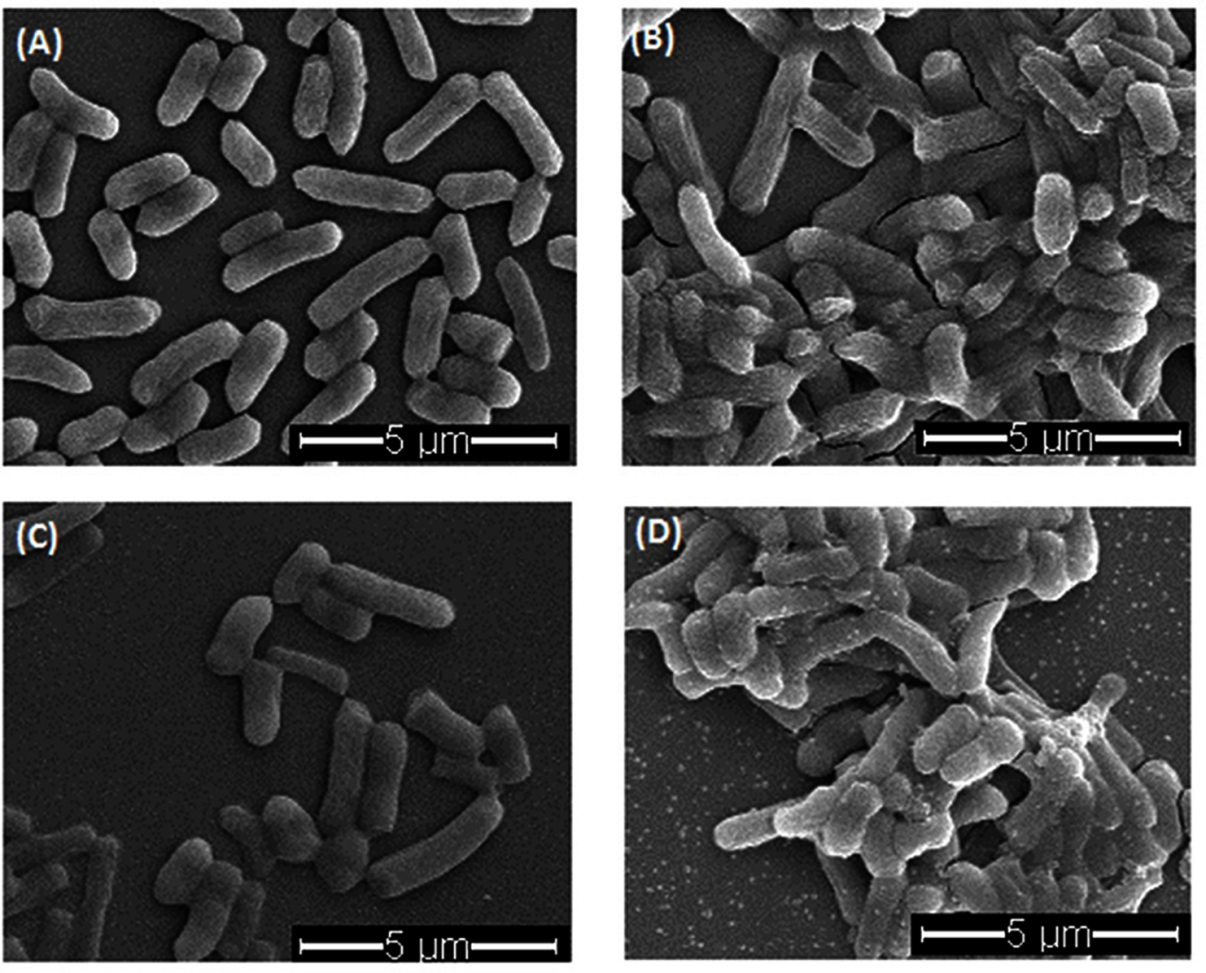
SEM images of *E*.*coli* cells with different treatments. (A) Untreated control samples; (B) CDots (10 μg/mL) treated samples; (C) H_2_O_2_ (8.82 mM) treated samples; (D) CDots (10 μg/mL) and H_2_O_2_ combination treated samples.

The SEM images of *E*.*coli* cells (Fig 5) indicated that part of the cells changed their morphologies after treated with 8.82 mM H_2_O_2_ alone or combined with 10 μg/mL CDots. The untreated cells were typically rod-shaped with intact cell walls, while some of H_2_O_2_ treated cells were thinner than and not as full as the untreated ones. Previous studies indicated that with H_2_O_2_ treatment, the morphology changes of *E*.*coli* cells were highly dependent on the H_2_O_2_ concentrations [33]. Treatment with low concentrations of H_2_O_2_ (< 2.5mM) could lead to an extensive cell filamentation at certain levels of H_2_O_2_ concentration or treatment time [33]. Both untreated or H_2_O_2_ alone treated cells were evenly distributed in the SEM images. As for the 10 μg/mL CDots treated bacteria, the morphologies did not show obvious change, but aggregation was observed in the SEM images. When the cells were treated with the combination of 8.82 mM H_2_O_2_ and 10 μg/mL CDots, cell sizes were slightly smaller than the untreated cells, and the aggregates were also formed. The aggregation with bacteria cells were common in many types of carbon based nanoparticles, such as single walled carbon nanotubes (SWCNTs), multi-walled carbon nanotubes, graphene oxide nanoparticles, etc. Some studies suggested that direct contact between cells and CNTs was essential for CNTs’ antimicrobial activities [34]. Although the aggregates formed between cells upon CDots treatment were tiny, the aggregation might related to the interactions between cells and CDots particles and to the antimicrobial efficacy of CDots alone and in combination with H_2_O_2_.

### Comparison with other CDots-antimicrobial reagents combination

Na_2_CO_3_ is another antimicrobial chemical, its antimicrobial mechanisms were primarily due to the changes in bacterial metabolisms induced by Na_2_CO_3_. In bacterial metabolism, many divalent cations serve as cofactors in many enzymes and can play important roles in the bacterial metabolisms [35]. CO_3_^2-^ is a divalent that can form complexes with a variety of divalent cations, such as Mg^2+^, Ca ^2+^, Zn^2+^, Fe^2+^, and these metal salts of carbonate are insoluble in alkaline pH. The insolubility of these metal salts formed by reacting with CO_3_^2-^ leads to the deficiency of the availabilities of metal cations for bacterial metabolisms. It was known that carbonate-treated cells had essentially no alkaline phosphatase activity which is a magnesium requiring enzyme and responsible for removing phosphate groups from many types of molecules, including nucleotides, proteins, and alkaloids [36]. Other studies reported treatment with CO^3-^ caused the release of periplasmic proteins by treated bacteria [35]. The distinctive property of carbonate in the removal of peripheral membrane proteins of low molecular weight has been reported in animal cells [37] and *E*. *coli* K-12 cells [38].

**Fig 6:**
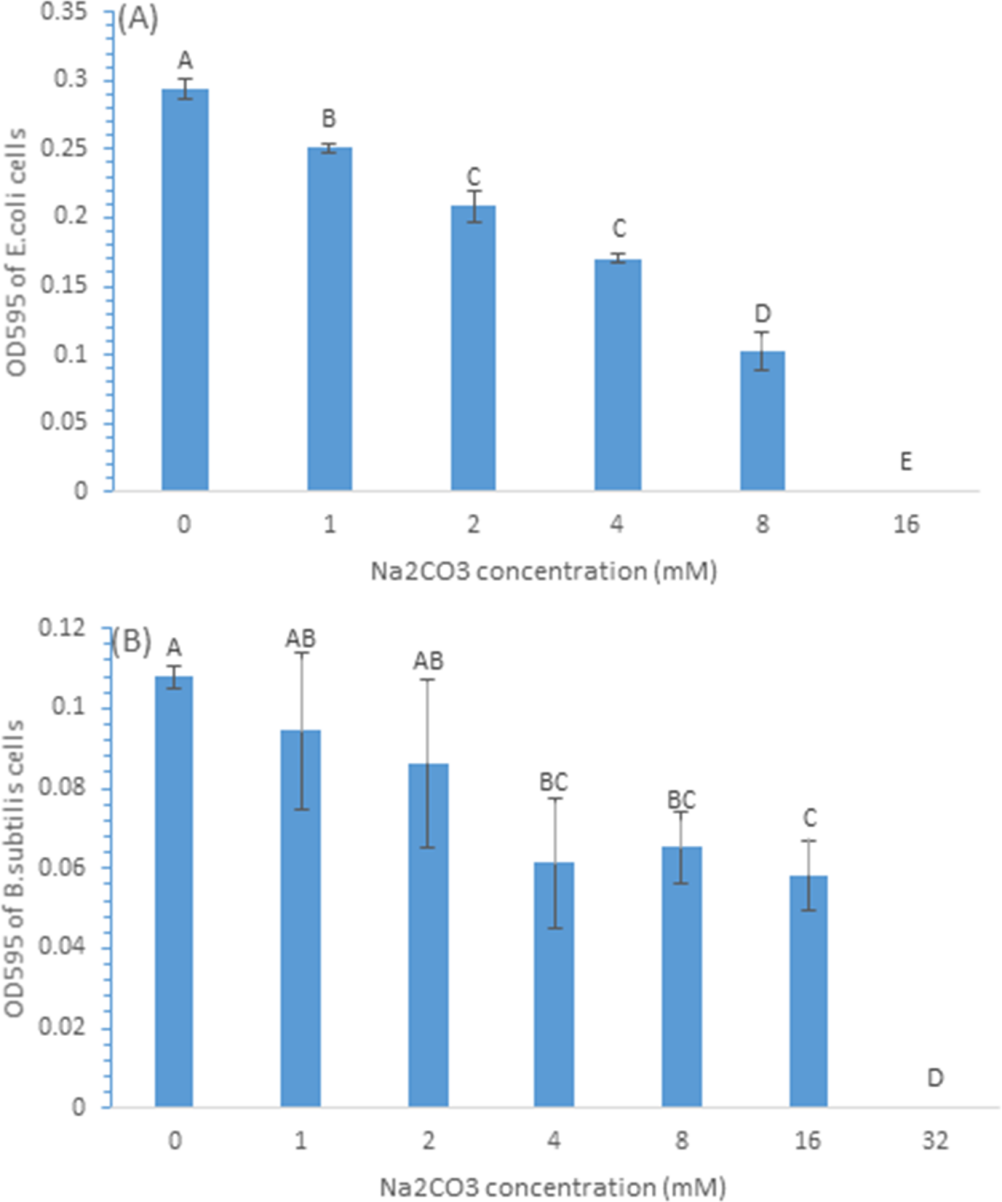
Antimicrobial effects of Na_2_CO_3_ alone on *E*.*coli* cells (A) and *B*.*subtilis* cells (B), and in combination with 10 μg/mL CDots (C: on *E*.*coli* cells). Different letters above the columns indicate statistically significant differences (p<0.05).

We first determined the MIC of Na_2_CO_3_ to *E*. *coli* and *B*. *subtilis* cells by examining the growth of the cells using OD measurement at 24 h of incubation after they were treated with different concentrations of Na_2_CO_3_. Fig 6 shows the inhibitory effect of Na_2_CO_3_ on *E*. *coli* and *B*. *subtilis* cells measured by OD after the cells were treated and incubated in the presence of a range of concentrations Na_2_CO_3_. With the increasing concentration of Na_2_CO_3_ from 0 to 16 or 32 mM, the inhibitory effect increased based on the decreased OD595 values. The MIC of Na_2_CO_3_ on *E*. *coli* cells and *B*. *subtilis* cells were 16 and 32 mM, respectively.

To investigate whether there was a synergistic effect of the combination treatment with Na_2_CO_3_ and CDots, inhibitory effect on the growth of *E*. *coli* and *B*. *subtilis* cells were performed using Na_2_CO_3_ and CDots alone or in combination using the microdilution checkboard method. Table 2 shows the combinations of different concentrations of Na_2_CO_3_ and CDots, and the growth statuses of the treated bacteria after 24 h incubation. “+” indicated there was bacterial growth, “-” indicated there was no bacterial growth.

**Table 2:** The combination of different concentrations of Na_2_CO_3_ and CDots used in the microdilution checkerboard method for treatments to E. coli cells, and the growth status of the cells at 24 h incubation after they were treated.

		CDots final concentration (μg/mL)								
		0	0.5	1	2	4	8	16	32	64
**Na**_**2**_**CO**_**3**_ **concentration (mM)**	0	+	+	+	+	+	+	+	+	-
	1	+	+	+	+	+	+	+	+	-
	2	+	+	+	+	+	+	+	+	-
	4	+	+	+	+	+	+	+	+	-
	8	+	+	+	+	+	+	+	+	-
	16	-	-	-	-	-	-	-	-	-

Table 3 Listed the MICs of the combination treatments based on the growth status observed in the results in Table 2 and the FIC index values of each component, and the ΣFICs of the combination treatments were calculated. As shown in Table 3, the calculated values of ΣFIC were in the range of 1.06–1.5, which indicated indifference relations in the combination treatments and no synergistic effect. Isobologram analysis also shows the line linking of MICs of the individual components and the line of the MICs of the component in the combination treatment were completely laid over, confirming no synergistic effect between the two components (S2 Fig).

**Table 3:** The MICs and FICs of individual agents in the combination treatments of CDots with H_2_O_2_, and the total FICs of combination treatments, obtained using the microdilution checkerboard method.

Combination Treatments		FIC of CDots	FIC of Na_2_CO_3_	ΣFIC
CDots (μg/mL)	Na_2_CO_3_ (mM)			
64 (MIC)	0	-	-	-
64	1	1	0.0625	1.0625
64	2	1	0.125	1.125
64	4	1	0.25	1.25
64	8	1	0.5	1.5
0	16 (MIC)	-	-	-
0.5	16	0.0078125	1	1.0078125
1	16	0.015625	1	1.015625
2	16	0.03125	1	1.03125
4	16	0.0625	1	1.0625
8	16	0.125	1	1.125

This is somewhat unexpected results, as it was expected that the detachment of peripheral proteins from bacteria membrane by CO_3_^2-^ and the photo-induced radical anions and cations by CDots could work together to enhance the antimicrobial effectiveness of the combination treatment. But the results revealed the fact that the two components did not work synergistically to enhance the overall antimicrobial function. Interestingly, synergistic antimicrobial relation between CO_3_^2-^ and other antimicrobial agents have been reported. Seathy et al. reported the antibacterial and antiviral activities of Ag^+^ can be enhanced ~ 1000 fold in presence of CO_3_^2-^ at the concentrations below the drinking water norms [39]. Sodium bicarbonate was also reported to have synergistic antibacterial effects when combined with antimicrobial agents ovotransferrin and tobramycin [40].

**Fig 7:**
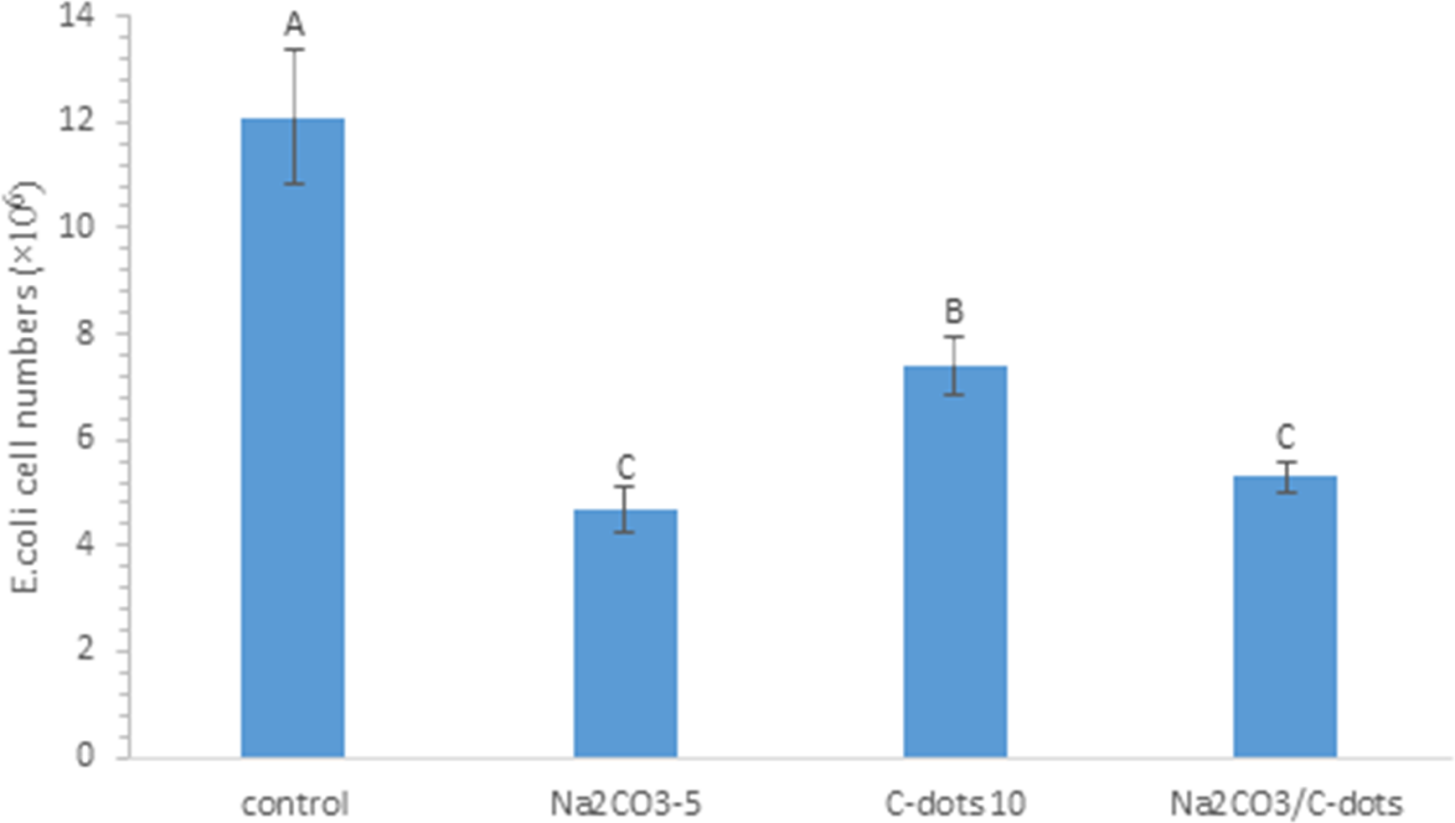
Antimicrobial effects of Na_2_CO_3_ in combination with 10 μg/mL CDots on *E*.*coli* cells. Different letters above the columns indicate statistically significant differences (p<0.05).

In a separated experiment with *E*. *coli* cells, the viable cell number was determined upon the treatments with Na_2_CO_3_, or CDots alone, and the combination of both. As shown in Fig 7, when *E*. *coli* cells were treated with 5mM Na_2_CO_3_ or 10 μg/mL CDots alone, the viable cell number decreased by 61.2% or 38.8%, respectively, while the treatment with the combination of 5 mM Na_2_CO_3_ and 10 μg/mL CDots only caused 56.2% decrease in viable cell numbers, which was less than the sum of the viable cell number reductions by the treatments with Na_2_CO_3_ alone and CDots alone, again confirming no synergistic effect in the combination of Na_2_CO_3_ and CDots.

Similar experiments were performed to examine whether there was synergistic effect of the combination treatment of CDots with another chemical AcOH. Table 4 shows the combinations of different concentrations of AcOH and CDots used in the microdilution method and the growth status of *E*. *coli* cells. The MIC of AcOH to E.coli cells was 1.18 mM. Table 5 shows the FICs of CDots and AcOH in the combination treatments, as well as the ΣFIC of the combination treatments, where their values were ~1.0 to 1.5, indicating there was indifference relations between the two components. Again, isobologram analysis (S3 Fig) showed the similar MIC line patter as shown in S2 Fig, confirming the indifference relation and no synergistic effect between CDots and AcOH.

**Table 4:** The combination of different concentrations of AcOH and CDots used in the microdilution checkerboard method for treatments to *E*. *coli* cells, and the growth status of the cells at 24 h incubation after they were treated.

		CDots final concentration (μg/mL)								
		0	0.5	1	2	4	8	16	32	64
**AcOH concentration (%)**	0	+	+	+	+	+	+	+	+	-
	0.000625	+	+	+	+	+	+	+	+	-
	0.00125	+	+	+	+	+	+	+	+	-
	0.0025	+	+	+	+	+	+	+	+	-
	0.005	+	+	+	+	+	+	+	+	-
	0.01	+	+	+	+	+	+	+	+	-
	0.02	+	+	+	+	+	+	+	+	-
	0.04	-	-	-	-	-	-	-	-	-

**Table 5:** The MICs and FICs of individual agents in the combination treatments of CDots with AcOH, and the total FICs of combination treatments, obtained using the microdilution checkerboard method.

Combination Treatments		FIC of CDots	FIC of AcOH	ΣFIC
CDots (μg/mL)	AcOH (%)			
64 (MIC)	0	-	-	-
64	0.000625	1	0.015625	1.015625
64	0.00125	1	0.03125	1.03125
64	0.0025	1	0.0625	1.0625
64	0.005	1	0.125	1.125
64	0.01	1	0.25	1.25
64	0.02	1	0.5	1.5
0	0.04 (MIC)	-	-	-
0.5	0.04	0.0078125	1	1.0078125
1	0.04	0.015625	1	1.015625
2	0.04	0.03125	1	1.03125
4	0.04	0.0625	1	1.0625
8	0.04	0.125	1	1.125
16	0.04	0.25	1	1.25
32	0.04	0.5	1	1.5

However, the indifference relations either between CDots and Na_2_CO_3_, or between CDots and AcOH, suggested that the changes of pH values in a certain range did not affect the antimicrobial effects of CDots since Na_2_CO_3_ provides basic environment and AcOH provide acidic environment in solution for CDots when interfaced with bacterial cells. This is an experimental observation that is consistent with the results reported by Sun et al. that CDots exhibited excellent stability in a wide pH range (pH 3–12), rendering them applicable in complicated and harsh conditions [41]. Another study reported that nitrogen-doped CDots exhibited pH-independent behavior in a wide pH range from 1 to 13 [42]. Compared to the combination of CDots with H_2_O_2_, it is still unclear at this stage why there is no synergistic effect in CDots combination with Na_2_CO_3_ or AcOH, but likely it might be due to insufficient ·OH free radical generated in the combination formulation to cause synergistic effect. However, further studies are necessary to investigate more detailed mechanisms on this.

## Conclusions

The results of this study indicated that the combination treatment with CDots and H_2_O_2_ exhibited synergistic effects to inhibit the growth of both Gram positive *B*. *subtilis* and Gram negative *E*. *coli* cell. However, the combination of CDots with Na_2_CO_3_ or AcOH exhibited no synergistic effects but indifference relations between the two components. Although the mechanistic understanding of the synergistic effect is not clear yet, such synergistic antimicrobial function of combination treatment could be a useful strategy to achieve the maximal inhibition of bacteria growth by using lower concentration of each chemical than that of individual component alone. As CDots are known to have very low to nontoxicity, CDots combinations with their synergistic antimicrobial reagents have the application potential to inhibit microbial growths more effectively, while reduce the risks imposed by other antimicrobial chemicals on environments and public health.

## Supporting information

S1 Fig E.coli cell growth curves after treated with H_2_O_2_ and CDots alone or in combination. The concentrations of H_2_O_2_ and CDots were 19.64 mM and 16 μg/mL, respectively.
(DOCX)

S2 Fig Isobologram of the interaction between CDots and Na_2_CO_3_ against *E*. *coli* cells. (TIF)

S3 Fig Isobologram of the interaction between CDots and AcOH against *E*. *coli* cells. (TIF)
