# Supplementary material for: Antibacterial effects of carbon dots in combination with other antimicrobial reagents
Source: PLoS One. 2017 Sep 21;12(9):e0185324. doi: 10.1371/journal.pone.0185324 (PMC5608398; doi:10.1371/journal.pone.0185324)
Supplement: S1 Fig — The concentrations of H2O2 and CDots were 19.64 mM and 16 μg/mL, respectively. (DOCX) [file pone.0185324.s001.docx]

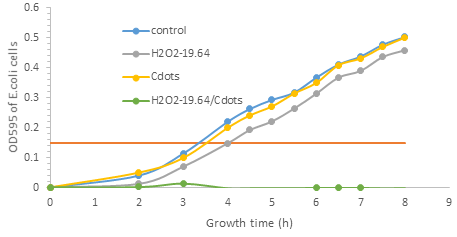


**S1 Fig. E.coli cell growth curves after treated with H_2_O_2_ and CDots alone or in combination. The concentrations of H_2_O_2_ and CDots were 19.64 mM and 16 µg/mL, respectively.**

Fresh overnight grown E.coli cells were diluted 5 time with PBS and the treatments with the final reaction volume of 200 µL were performed in a 96-well plate under light for 1 h. Each reaction was then added to 3.8 mL LB broth and incubated at 37˚C in a shaker at 225 rpm for 8 h. OD595 values were read at a series time point and pH values were also monitored. After 8 h incubation, pH values were changed from 6.83 to 7.60, 7.28, 7.58, and 7.11 for the control sample, H_2_O_2_ treated alone, CDots treated alone, and H_2_O_2_/CDots treated samples, respectively, due to the cell growths.
