# Supplementary figures and images for: Antibacterial effects of carbon dots in combination with other antimicrobial reagents
Source: PLoS One. 2017 Sep 21;12(9):e0185324. doi: 10.1371/journal.pone.0185324 (PMC5608398; doi:10.1371/journal.pone.0185324)

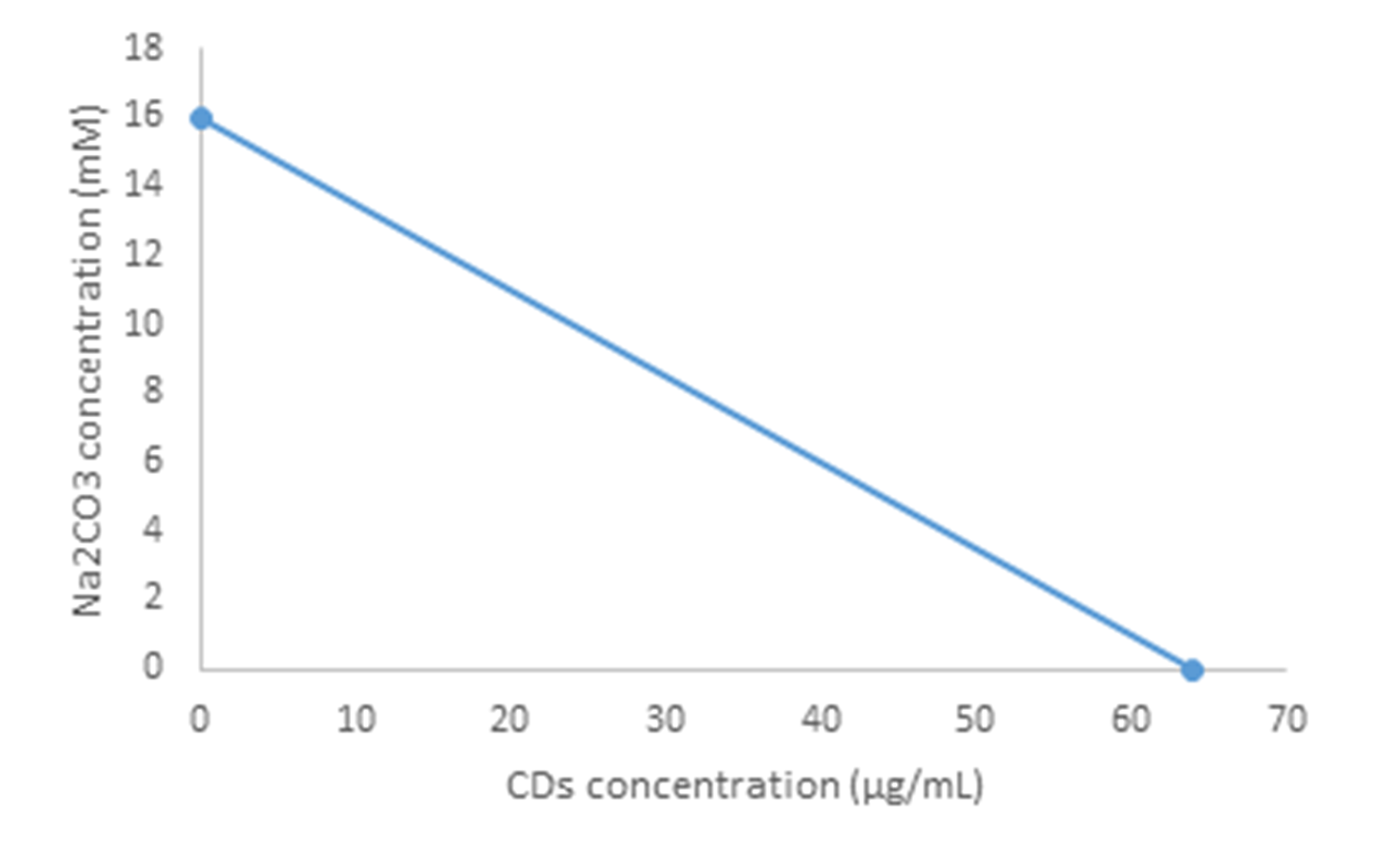

Supplement: S2 Fig — (TIF) [file pone.0185324.s002.tif]

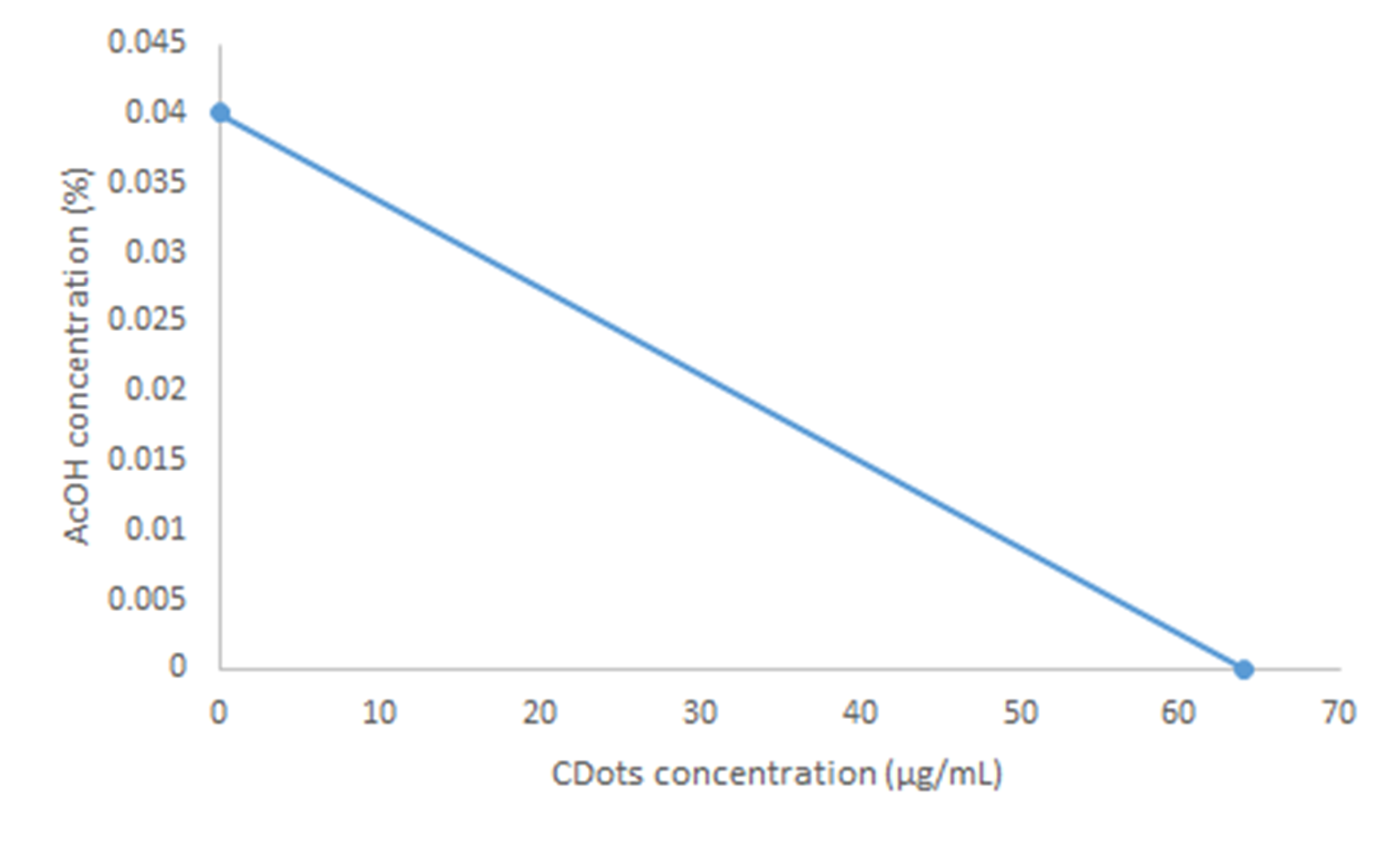

Supplement: S3 Fig — (TIF) [file pone.0185324.s003.tif]
